# Identification of aaNAT5b as a spermine N-acetyltransferase in the mosquito, *Aedes aegypti*

**DOI:** 10.1371/journal.pone.0194499

**Published:** 2018-03-19

**Authors:** Huai Guan, Maoying Wang, Chenghong Liao, Jing Liang, Prajwalini Mehere, Meiling Tian, Hairong Liu, Howard Robinson, Jianyong Li, Qian Han

**Affiliations:** 1 Key Laboratory of Tropical Biological Resources of Ministry of Education, Hainan University, Haikou, Hainan, China; 2 Laboratory of Tropical Veterinary Medicine and Vector Biology, Institute of Tropical Agriculture and Forestry, Hainan University, Haikou, Hainan, China; 3 Krantisinh Nana Patil College of Veterinary Science, Shirval, India; 4 Department of Biochemistry, Virginia Tech, Blacksburg, Virginia, United States of America; 5 Biology Department, Brookhaven National Laboratory, Upton, New York, United States of America; Universidade Federal do Rio de Janeiro, BRAZIL

## Abstract

Mosquitoes transmit a number of diseases in animals and humans, including Dengue, Chikungunya and Zika viruses that affect millions of people each year. Controlling the disease-transmitting mosquitoes has proven to be a successful strategy to reduce the viruses transmission. Polyamines are required for the life cycle of the RNA viruses, Chikungunya virus and Zika virus, and a depletion of spermidine and spermine in the host via induction of spermine N-acetyltransferase restricts their replication. Spermine N-acetyltransferase is a key catabolic enzyme in the polyamine pathway, however there is no information of the enzyme identification in any insects. Aliphatic polyamines play a fundamental role in tissue growth and development in organisms. They are acetylated by spermidine/spermine *N*_*1*_-acetyltransferase (SAT). In this study we provided a molecular and biochemical identification of SAT from *Aedes aegypti* mosquitoes. Screening of purified recombinant proteins against polyamines established that aaNAT5b, named previously based on sequence similarity with identified aaNAT1 in insects, is active to spermine and spermidine. A crystal structure was determined and used in molecular docking in this study. Key residues were identified to be involved in spermine binding using molecular docking and simulation. In addition, SAT transcript was down regulated by blood feeding using a real time PCR test. Based on its substrate profile and transcriptional levels after blood feeding, together with previous reports for polyamines required in arboviruses replication, SAT might be potentially used as a target to control arboviruses with human interference.

## Introduction

The naturally occurring aliphatic polyamines classically comprise three molecules (putrescine, spermidine and spermine) and recently agmatine was also included. Polyamines are known to play a fundamental role in tissue growth and development, and their physiological importance was documented in numerous studies [1]. Therefore, in-cell polyamine homeostasis is tightly controlled at key steps of cell metabolism. It is well-known that ornithine decarboxylase (ODC, a rate-limiting enzyme for polyamine biosynthesis) activity and polyamine levels increase during tissue proliferation and decline as growth processes cease. Most polyamines are derived from the amino acids ornithine and methionine. ODC converts L-ornithine to putrescine, the initial step in the biosynthesis of polyamines. Subsequently, aminopropyl groups are added to putrescine from the methionine derivative, decarboxylated S-adenosylmethionine, to form spermidine and spermine catalyzed by polyamine synthases. In addition to their synthesis, polyamine levels and activity are regulated by their catabolism. Polyamines can be acetylated by spermidine/spermine N-acetyltransferase (SAT) and/or oxidized by polyamine oxidases and acetylated polyamine oxidases [2]. Because polyamines affect so many cell processes, the enzymes involved in polyamine synthesis and catabolism are tightly regulated for their expression and activities. Steady-state levels of polyamines are maintained by regulation of ODC activity to alter polyamine synthesis, as well as through polyamine catabolic enzymes, including SAT which promotes their interconversion with putrescine or export from the cell [3].

Interestingly, polyamines are also involved in host-pathogen interactions. Recently it was reported that polyamines are required for the life cycle of the RNA viruses Chikungunya virus (CHIKV) and Zika virus (ZIKV). Depletion of spermidine and spermine in the host via induction of SAT, a key catabolic enzyme in the polyamine pathway, restricts CHIKV and ZIKV replication [4].

Although the enzymes involved in polyamine synthesis and catabolism are conserved across all kingdoms of life [5], only a few studies have concerned polyamines in insects. In female crickets, it was reported that the oviposition behavior is changed after polyamines were depleted in the bodies (Myriam Cayre, 1996). In silkworms, *Bombyx mori* polyamine treatment would influence silk quality at structural, mechanical, and molecular level, which can be exploited in silk biomaterial production [6]. *Aedes aegypti* and *Ae*. *albopictus* transmit dengue virus, CHIKV and ZIKV. Since CHIKV and ZIKV replications need polyamines [4], depletion of polyamines from mosquitoes may prevent transmission of such viruses. However, no enzymes have been biochemically identified to be involved in polyamine synthesis and catabolism in any insects, which is an obstacle to use any molecular approach to manipulate polyamine levels in mosquitoes. Particularly, the enzyme, SAT that acts as a viral restriction factor of CHIKV and ZIKV [4] has not been reported in mosquitoes. In order to understand effects polyamines on arboviruses replication, it is necessary identify the gene coding for SAT in mosquitoes.

In this study, we over-expressed aaNAT5b in a bacterial protein expression system and screened the polyamine substrates for the recombinant protein, and obtained a new crystal structure for further structure and enzyme molecular mechanism study. The results obtained in this study will provide basic information for further validating, if the polyamine biosynthetic pathway is an attractive target for the development of antivirals in mosquitoes.

## Materials and methods

### Expression and purification of aaNAT5b from *Ae*. *aegypti*

All chemicals were purchased from Sigma-Aldrich (St. Louis, MO, USA) unless otherwise described. According to previous methods [7,8], amplification of cDNA for *aaNAT5b* (VectorBase Gene ID: AAEL004827, GeneBank accession no: CH477317.1) was achieved using the forward and reverse primers corresponding to 5’- and 3’-regions of the coding sequences. The amplified cDNA sequence was cloned into an Impact^™^-CN plasmid (New England Biolabs) for expression of its recombinant protein. *Escherichia coli* BL21 (DE3) cells (Promega, Madison, WI, USA**)** transformed with *aaNAT5b* recombinant plasmid were cultured at 37 °C. The cells were cultured at 15 °C for 24 hours after induction with 0.1 mM isopropyl-1-thio-β-D-galactopyranoside (IPTG). Five liters of cell cultures were used for the expression of the recombinant protein [7]. The recombinant proteins were purified and concentrated for further biochemical and crystallization studies according to previous methods [7,8]. The purity of the *aaNAT5b* was assessed by the presence of a single band in correct size on an SDS-PAGE(sodium dodecyl sulfate–polyacrylamide gel electrophoresis). The protein concentration was determined by a Bio-Rad protein assay kit using bovine serum albumin (Sigma-Aldrich, St. Louis, MO, USA) as a standard.

### SAT activity assays

SAT activity assay was based on the hydrolysis of Acetyl-CoA in the presence of polyamine substrates by the enzyme. 5′-dithio-bis(2-nitrobenzoic acid) (DTNB) was used as a colorimetric developing agent for detection of CoA. The substrate (2 mM) [9] and 0.4 μg purified recombinant aaNAT5b were mixed and pre-incubated at room temperature for 5 min in a 250 μL 96-well plate. Acetyl-CoA (0.4 mM) was added to start the reaction in a final volume of 100 μL. The final mixture contained 50 mM Tris-HCl, and 1.0 mg mL^-1^ bovine serum albumin (for stabilizing the enzymes). The mixture was continually incubated at room temperature for 5 min. The reaction was stopped with 25 μL guanidine hydrochloride solution (6.4 M guanidine-HCl, 0.1 M Tris-HCl, pH 7.3) containing 5 mM DTNB and the absorbance at 405 nm was measured on a plate-reader within 5 min. Reactions wherein polyamines, Acetyl-CoA or aaNAT5b was omitted respectively were used as controls. The amount of CoA produced was determined from a standard curve generated using CoA standards. N1-acetylspermine was also tested for a possible substrate to clarify if both free amine groups are able to accept acetyl group from acetyl-CoA.

### Kinetic analysis

SAT was active to polyamines, including spermine, spermidine, putrescine and agmatine and their kinetic properties to both polyamines were assessed further by measuring their specific activity in the presence of varying concentrations (0.016 to 2 mM) of each polyamine and a fixed concentration of acetyl-CoA (0.4 mM). For a comparison purpose, previous reported substrate histamine and hydrazine [8] were also tested for their kinetic characteristics. The data were fitted to the Michaelis–Menten equation by the non-linear regression method. Estimation of apparent K_m_ values was obtained by SigmaPlot Enzyme Kinetics Module (SPSS, San Jose, CA). Each substrate was tested in triplicate. Data are the means ± SEM of three experiments.

### Protein crystallization and structural determination

The crystals were grown by a hanging-drop vapor diffusion method with the volume of reservoir solution at 500 μl and the drop volume at 2 μl, containing 1 μl of protein sample and 1 μl of reservoir solution [8]. The crystallization buffer is consisted of 0.5 M sodium chloride, 25% polyethylene glycol 8000, 15% glycerol, and 0.1 M sodium acetate (pH 4.6). Crystals were soaked in the crystallization buffer, which contains 0.5 mM hydrazine for 2 min before being cryogenized in the crystallization buffer.

Diffraction data of the crystal was collected at the Brookhaven National Synchrotron Light Source beam line X29A (λ = 1.075 Å). Data were collected using an ADSC Q315 CCD detector. The structure was determined by the molecular replacement method using the published mosquito aaNAT5b structure (Protein Data Bank code, 4fd4) [8]. The program Molrep [10] was employed to calculate both cross-rotation and translation of the model. The initial model was subjected to iterative cycles of crystallographic refinement with the Refmac 5.3 [11] and graphic sessions for model building using the program Coot 0.7.1 [12]. The cofactor molecule was modeled when the R factor dropped to a value of around 0.21 at full resolution for the structures, based upon both the 2Fo–Fc and Fo–Fc electron density maps. Solvent molecules were automatically added and refined with ARP/warp [13] and Refmac 5.3.

### Molecular docking and dynamics simulation

The crystal structure model was used for molecular docking. AutoDock Vina [14] was utilized to produce active site-substrate molecular docking solutions for the SAT crystal structure using four aliphatic polyamines, hydrazine and histamine as ligands. The ligands and receptor were prepared by AutoDock Tools 1.5.6 (http://mgltools.scripps.edu/). The side chains of residues around the active-site cavity were set flexible and the rotatable bonds of ligands were left free to rotate. The grid box (35×20 ×24Å) covered the active-site cavity. Pymol [15] was then used to visualize the active site residues within 5 Å of the docking solution with the highest (kcat/mol) affinity from five docking experiments. The residues proximal to the ligands from the crystal structure were identified potential substrate binding or specifying residues.

The stability of the protein-ligand complexes revealed by the docking experiment was validated using molecular dynamics (MD) simulation by Gromacs 5.1.4 [16]. The topology and parameter files for aaNAT5b were produced from AmberTools 16 (University of California, San Francisco) program with the Generalized Amber Force Field (GAFF) and ACPYPE [17]. The Amber99SB force field was applied to the protein structure. The complex was surrounded by TIP3P water molecules and system was neutralized with Na^+^ ions. The dodecahedron periodic water boxes with a margin of 1.0 nm were used for MD simulation. The solvated structure was minimized by the steepest descent method until maximum force reaches < 1000.0 kJ/mol/nm at 300K temperature and constant pressure. Then the complex was equilibrated for 200 ps constant number of particles, volume, and temperature (NVT) condition and 200 ps constant number of particles, pressure, and temperature (NPT) condition at 1 atm pressure and 300K, each step 2 fs. LINCS algorithm was utilized for covalent bond constraints in the equilibration steps. For the calculation of Lennard-Jones and Coulomb interactions, 1.4 nm radius cut-off was used. Long range electrostatics was calculated by using Particle Mesh Ewald (PME) method with Fourier grid spacing of 1.6 Å. The temperature inside the box was regulated by using V-rescale, a modified Berendsen temperature coupling method. Parrinello-Rahman pressure coupling method was utilized in NPT equilibration. 20 ns production MD was run after equilibration at 1 atm and 300K under the NPT ensemble with a time step of 2 fs. Trajectories were saved and results were analyzed using XMGRACE.

### Expression profiling of aaNAT5b based on real time quantitative reverse transcriptase PCR (qRT-PCR)

qRT-PCR was performed to compare the transcriptional levels of aaNAT5b from adult female abdomens, thoraxes, and heads of blood fed and sugar fed mosquitoes. RNA isolation and cDNA synthesis for the qRT-PCR were done according to a previous report [7]. Briefly, samples of abdomens, thoraxes, and heads were collected from sugar fed and blood fed mosquitoes after 48 hours of feeding, and ovary samples were collected from sugar fed mosquitoes after 48 hours of feeding. Total RNA of each sample was extracted and treated with DNase I (Promega) to remove the contaminating DNAs. Then about 5 μg of total RNA was reverse transcribed into cDNA using a GoScript reverse transcription system (Promega). The synthesized cDNA was diluted (1: 5) before the qRT-PCR test and stored at -20 °C until used. A primer pair that amplifies a fragment of *Ae*. *aegypti* glyceraldehyde-3-phosphate dehydrogenase (*gapdh*) gene, a relatively abundant and constitutively expressed gene, was used to normalize the results of variable target genes and to correct for sample-to-sample variations [18]. qRT-PCR was performed in a LightCycler 480 system (Roche Applied Science, Mannheim, Germany) using SYBR green Master I (Roche) according to manufacturers’ instructions, with the following cycling conditions: 95 °C for 10 min, followed by 45 cycles of 95 °C for 10 s, and 55 °C for 10 s,72 °C for 10 s. All test samples and the controls were performed in triplicate. The comparative 2^-ΔΔct^ method was used to quantify aaNAT5b transcripts. All the samples were normalized with respect to *gapdh*. All data were tackled using Excel and showed as mean ± SD (*n* = 3). One-way ANOVA and multiple comparisons were performed by SPSS 19.0 (IBM, USA), and *p* < 0.05 was considered significant difference.

## Results

### Bioinformatic information of putative spermine acetyltransferase proteins from Ae. aegypti

Human spermidine/spermine N1-acetyltransferase 1 (SAT1, NCBI Reference Sequence: NP_002961.1), which is capable of catalyzing the acetylation of spermidine and spermine [19] was used as a query to search the *Ae*. *aegypti* genomic sequences in the National Center of Biotechnology and Information (NCBI) database using the BLAST and PSI BLAST search programs [20], no meaningful homologue was found. However, thirteen individual sequences obtained from *Ae aegypti* genome sequences based on a BLAST search using *Drosophila* arylalkylamine *N*-acyltransferase (aaNAT1) as a query [8] contain two motifs (commonly named Motif A and Motif B), which are characteristic of the *N*-acyltransferase superfamily [21]. Phyletic distribution analysis confirmed three major clusters, and one of them was called polyamine N-acetyltransferase like cluster [8]. CLUSTAL W analysis [22] shows human SAT1 shares 5–13% sequence identity with the reported thirteen *Ae*. *aegypti* aaNAT and putative aaNAT sequences. The available information is apparently not quite enough to propose which *Ae*. *aegypti* aaNAT and putative aaNAT is a SAT protein. Biochemical test of individual proteins is necessary to identify mosquito SAT.

### Protein expression and purification, substrate specificity and kinetic analysis of aaNAT5b

There are three reasons why we screened aaNAT5b for its SAT activity at first: 1) it can acetylate histamine and hydrazine, which are structurally similar to polyamines; 2) it was proposed to be in polyamine N-acetyltransferase like cluster by phylogenetic analysis; and 3) its crystal structure was available for further structural analysis [8]. According to a previous reported method, aaNAT5b coding sequence was amplified from *Ae*. *aegypti* cDNA preparations and its recombinant protein was expressed using the bacterial protein expression system. Soluble recombinant protein of aaNAT5b was purified sequentially by chitin affinity, ion exchange, and gel filtration chromatography. The final preparation contained the major single band for aaNAT5b around 25 kDa. The recombinant protein was screened for SAT activity using spermine, spermidine, putrescine and agmatine as the acetyl group acceptor and acetyl-CoA as the acetyl group donor. Although aaNAT5b recombinant protein displayed SAT activity to all tested substrates, spermine and agmatine are much better substrates than others. Its affinity and catalytic efficiency to four aliphatic polylamines and two previously reported substrates (histamine and hydrazine) were tested. The kinetic parameters are given in Table 1. When N_1_-acetylspermine was used as a substrate in the same reaction mixture as done for SAT activity assay using spermine as a substrate, no acetylation activity was found, suggesting only one free amine group or the N_1_ atom is able to accept acetyl group from acetyl-CoA.

**Table 1:** Kinetic analysis of *Ae*. *aegypti* aaNAT5b/SAT. The activities were measured as described in the materials and methods section. Each substrate was tested in triplicate. The parameters were calculated by fitting the Michaelis–Menten equation to the experimental data using the enzyme kinetics module in SigmaPlot. Results are means ± SE.

	K_m_	*k*_*cat*_	*k*_*ca*t_ /K_m_
	mM	min^-1^	min^-1^mM^-1^
SAT /aaNAT5b			
Spermine	0.17 ± 0.04	199.1±12	1171
Agmatine	0.21 ± 0.05	150.2 ± 11	715
Hydrazine	0.26 ± 0.03	38.2 ± 1.7	147
Putrescine	0.52 ± 0.11	30.2 ± 1.6	58
Histamine	0.70 ± 0.13	16.6 ± 1.3	24
Spermidine	0.69 ± 0.12	15.6 ± 1.4	23

### Overall structures of aaNAT5b/SAT

**Fig 1:**
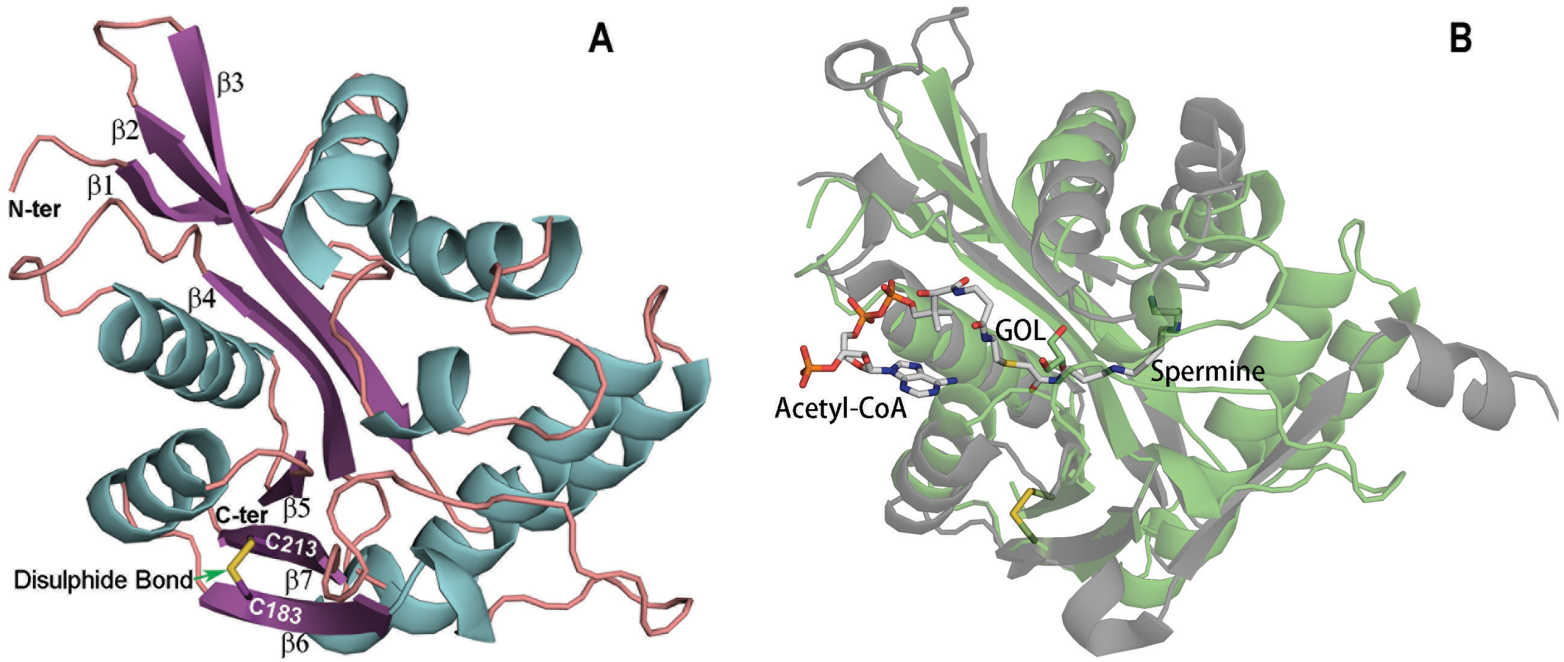
A schematic view of the aaNAT5b/SAT structure and comparison with human SAT. The molecular structures of proteins are shown in cartoon presentation. A disulphide bond, the molecules glycerol (GOL), acetyl-CoA, spermine are shown in stick view. β strand number of aaNAT5b is labeled in (A); and human SAT and aaNAT5b are colored gray and green, respectively in (B).

In an effort to investigate the structure-function relationship of mosquito SAT enzyme and the substrate selectivity, efforts were made to crystallize its native enzyme and to soak them with its substrates or substrate analogues to determine its complex structure. After extensive optimizations, X-ray diffraction data was collected from the crystal of aaNAT5b soaked with hydrazine. The structure was determined by molecular replacement using a previously reported aaNAT5b native structure (Protein Data Bank code, 4fd4) [8] as a search model. The data collection, refinement statistics, and statistics on Ramachandran plots as analyzed using Molprobity [23] of the structure are provided in Table 2. The overall architecture of the structure is the same as in our previous report, with RMSD values of 0.1 to 0.5 Å between chains [8]. However, a disulphide bond between residue 183 and residue 213 was found in the structure and it links two β strands (β7 and β6) (Fig 1A). In addition, residues 1–5 were modeled in the new complex structure, whereas they were not modeled in the previously reported structure because of the poor electron density in the region [8]. By superposing the structure onto human SAT complex structure (PDB code, 2jev) [24], we found aaNAT5b/SAT structure share similar architecture with human SAT, particularly in the cofactor binding region (Fig 1B).

**Table 2:** Data collection and refinement statistics for the aaNAT5b/SAT complex.

Crystal data	aaNAT5b /SAT
Space group	P212121
Unit cell	
a, Å	38.3
b, Å	72.9
c, Å	149.7
α = β = γ	90
Data collection	
X-ray source	BNL-X29*
Wavelength, Å	1.075
Resolution, Å†	1.95 (1.998–1.948)
Total number of reflections	364, 955
Number of unique reflections	31,303
R_merge_^†^	0.12 (0.50)/
*I/σI*	21.0 (4.0)/
Redundancy^†^	11.7 (12.0)/
Completeness, %^†^	99.8 (100.0)/
Refinement statistics	
Rwork, %	20.3
Rfree, %	25.2
RMS bond length, Å	0.007
RMS bond angle, degrees	1.081
No. of ligand molecules	
Glycerol	7
No. of water molecules	188
Average B value, Å^2^	39.0
Average B value, Glycerol, Å^2^	48.3
Average B value, Water, Å^2^	42.6
Statistics on Ramachandran plot, %	
Favored regions	98.1
Generously allowed regions	1.9

*BNL, Brookhaven National Laboratory.

^†^Values in parentheses are for the highest-resolution shell.

### Polyamine substrate binding revealed by molecular docking and simulation

**Fig 2:**
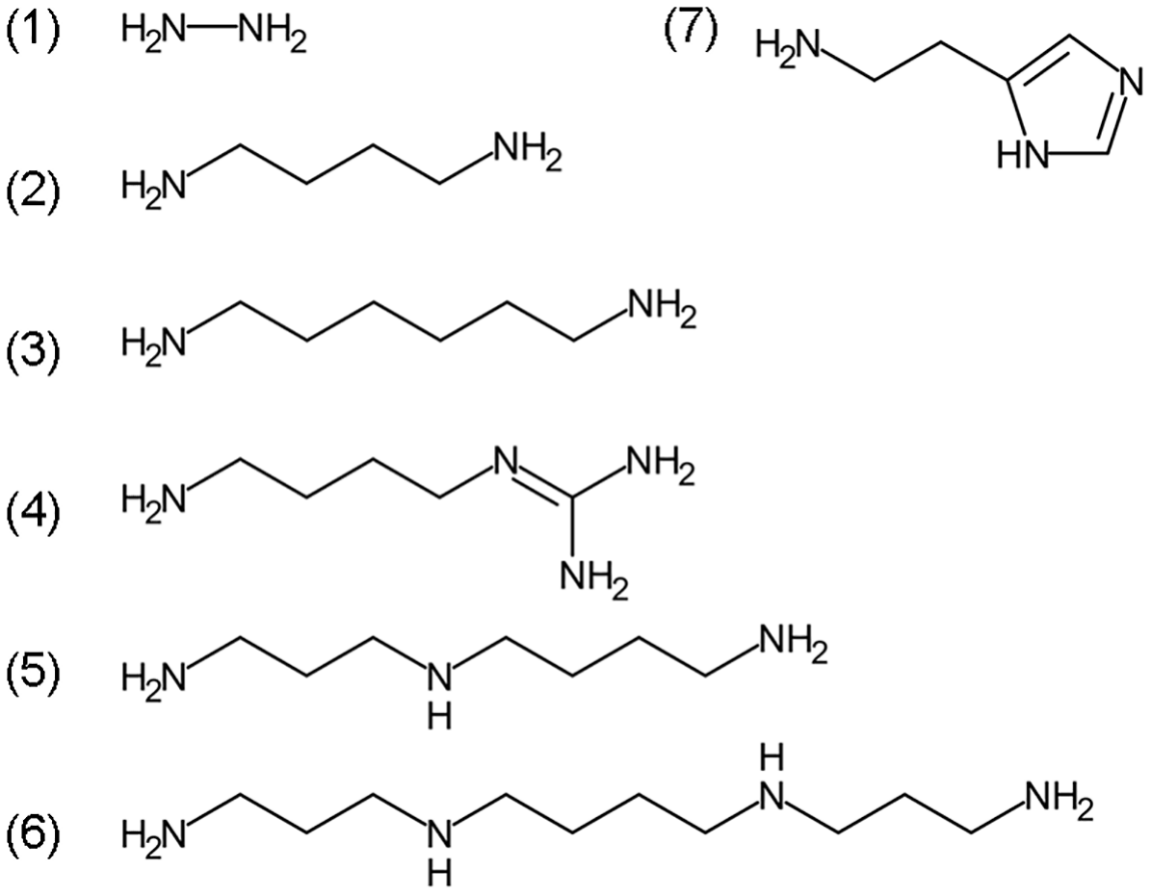
The chemical structure of ligands. (1) Hydrazine, (2) Putrescine, (3) Hexmethylenediamine, (4) Agmatine, (5) Spermidine, (6) Spermine, and (7) Histamine.

**Fig 3:**
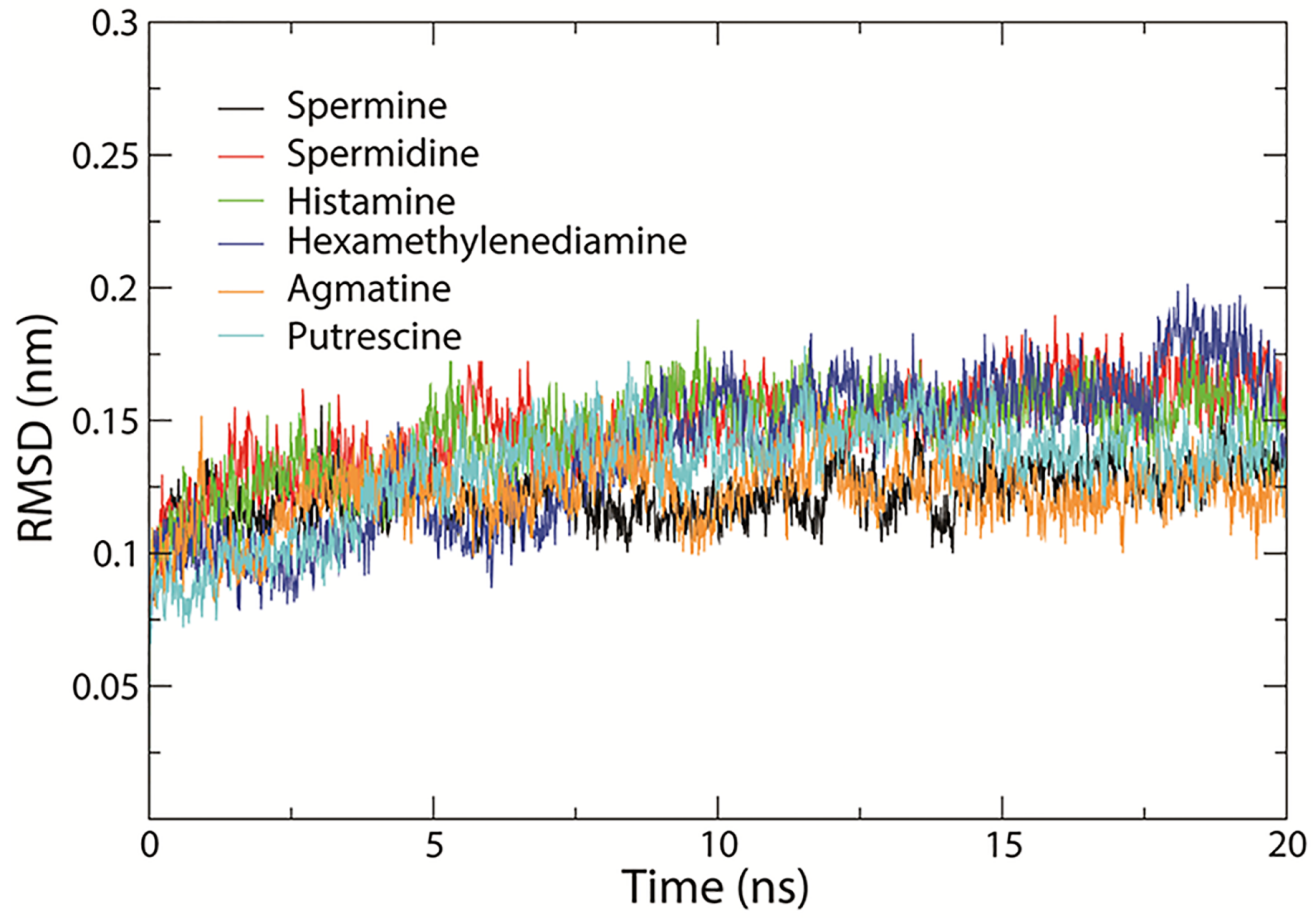
Backbone RMSD of the structures during 20 ns simulation. The ordinate is RMSD (nm), and the abscissa is time (ns). Black, red, green, blue, orange, and cyan lines indicate the structure of aaNAT5b with ligands: spermine, spermidine, histamine, hexamethylenediamine, agmatine, and putrescine, respectively.

**Fig 4:**
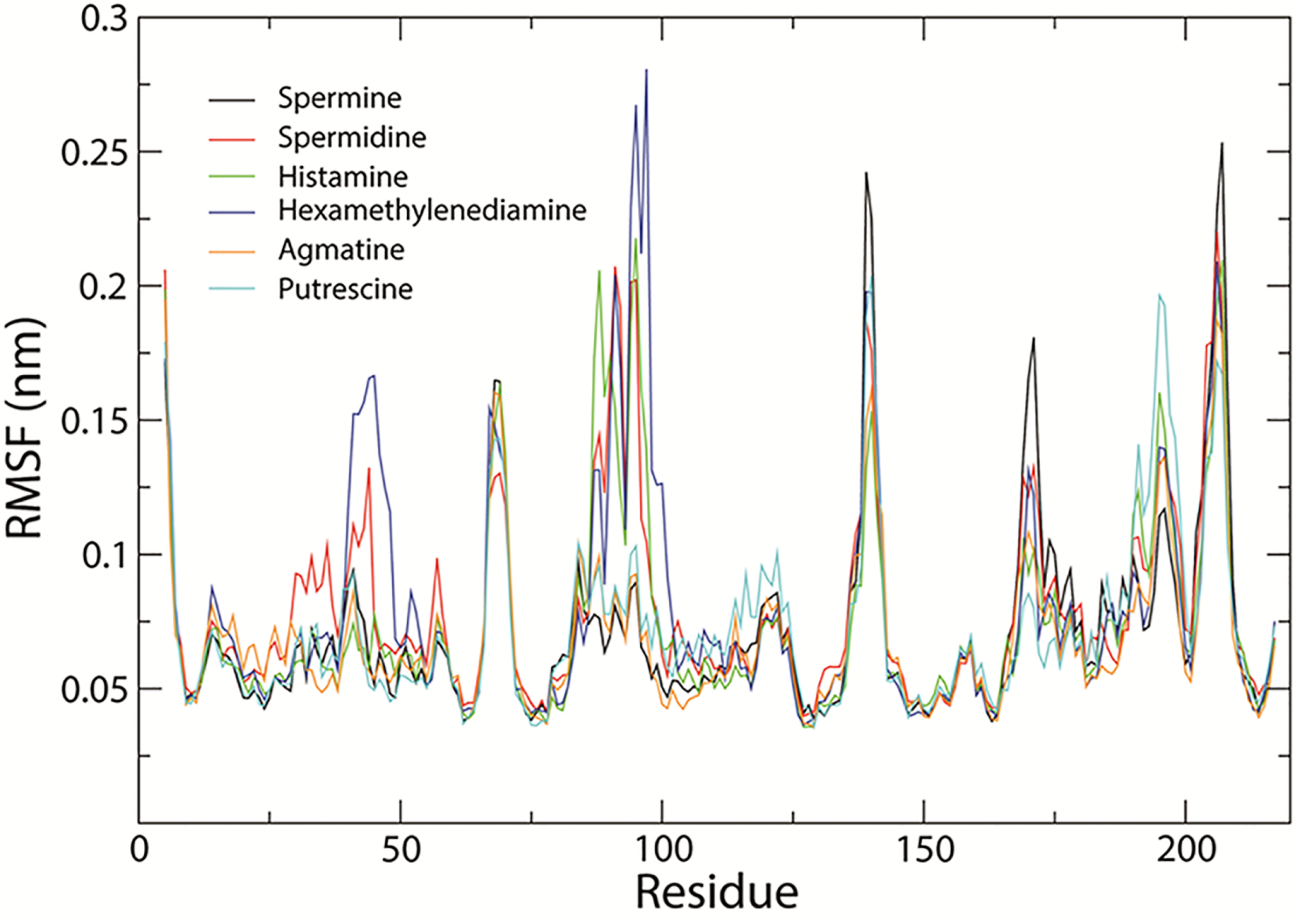
Carbon alpha RMSF of the structures of aaNAT5b with ligands during in 20 ns simulation. The ordinate is RMSF (nm), and the abscissa represents the residues. Black, red, green, blue, orange, and cyan lines indicate the the structure of aaNAT5b with spermine, spermidine, histamine, hexamethylenediamine, agmatine, and putrescine, respectively.

**Fig 5:**
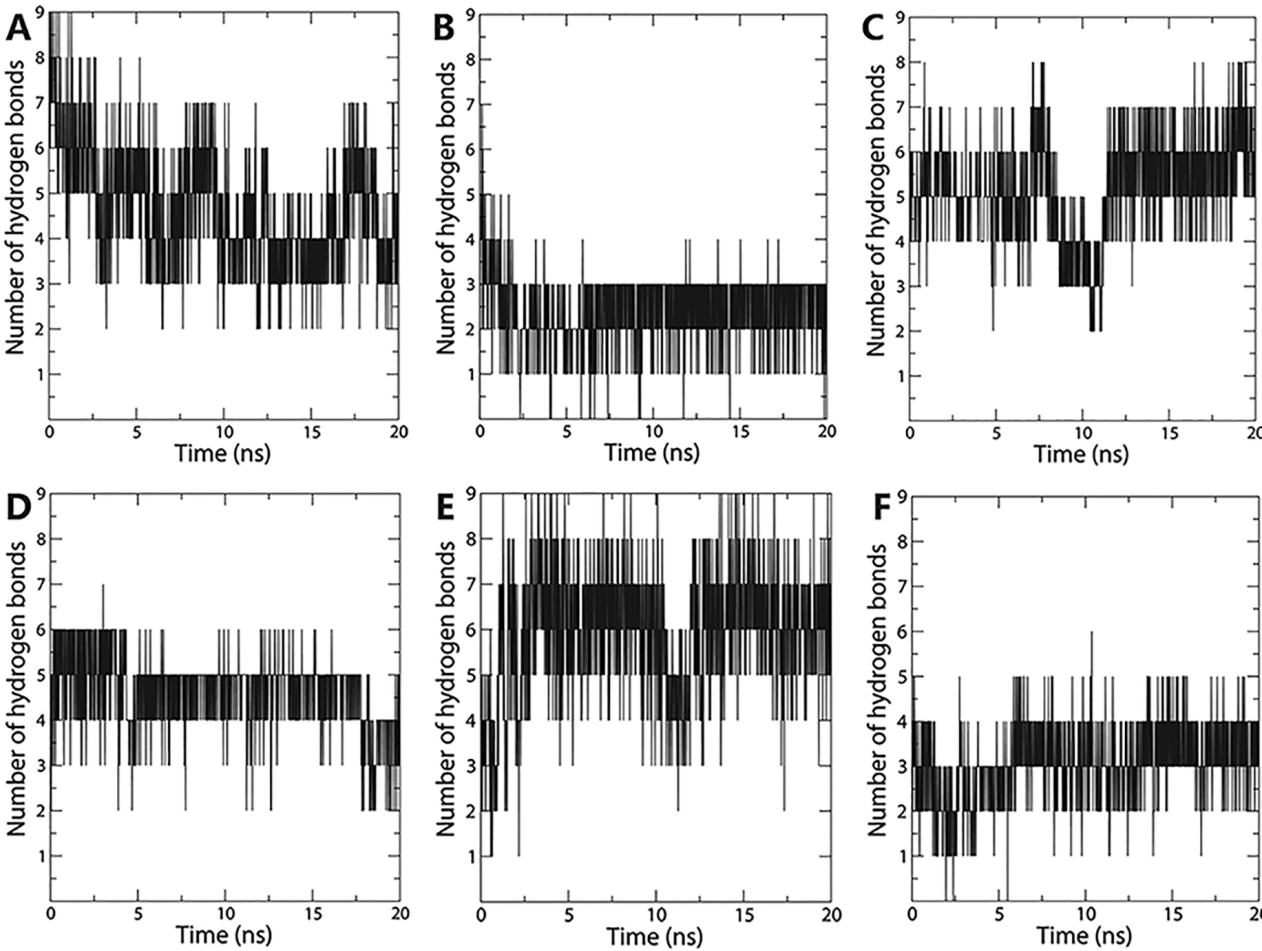
Number of hydrogen bond formed in ligand and aaNAT5b. The ordinate is the number of hydrogen bond and the abscissa is time (ns). (A) spermine, (B) histamine, (C) spermidine, (D) hexamethylenediamine, (E) agmatine, and (F) putrescine.

**Fig 6:**
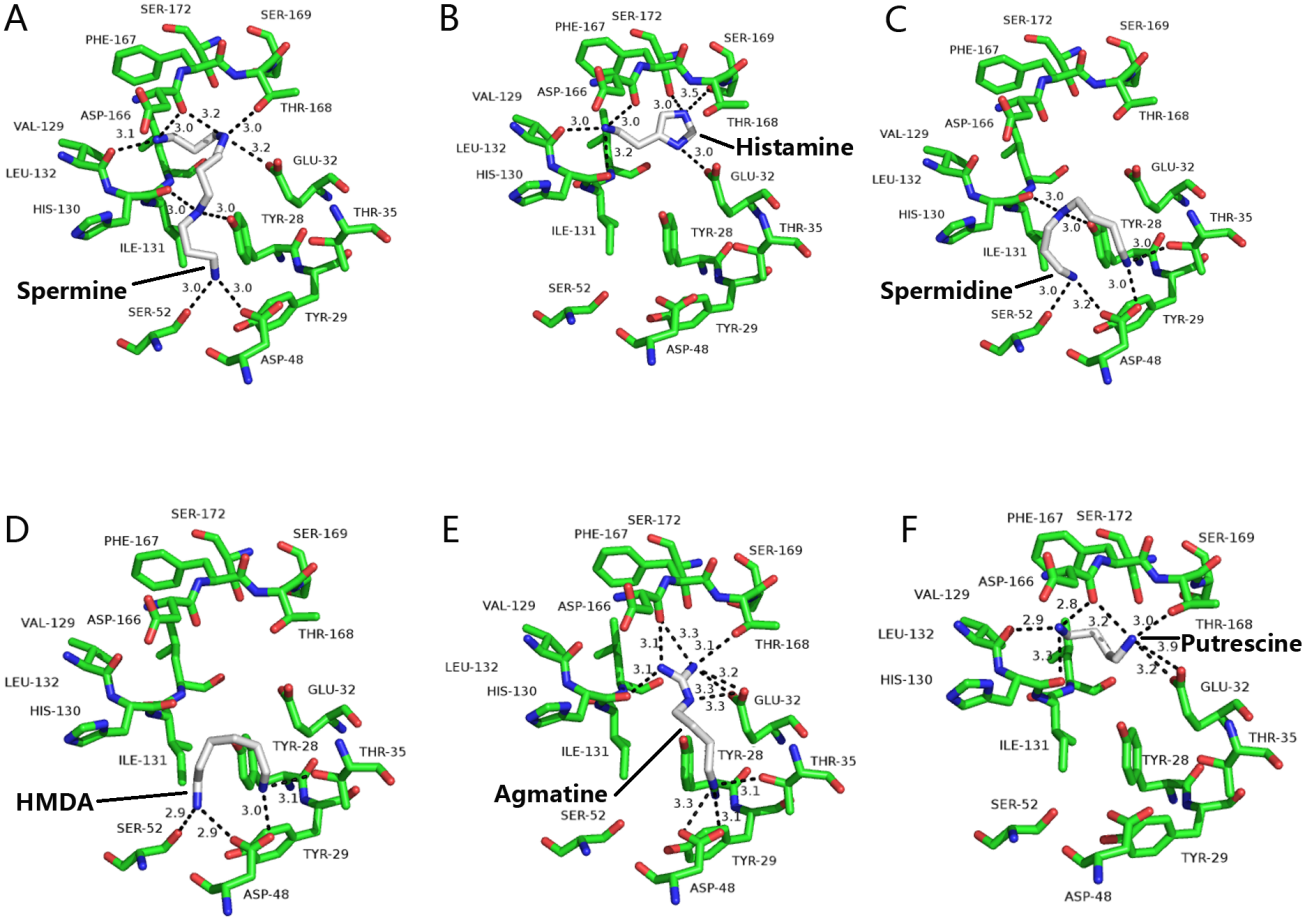
Docked complexes showing interactions between substrates and aaNAT5b. The ligands, (A) spermine, (B) histamine, (C) spermidine, (D) hexamethylenediamine (HMDA), (E) agmatine, and (F) putrescine are shown in stick presentation and colored for different elements (grey for carbon atom and blue for nitrogen atom). Residues interacting with the ligand are shown in stick presentation too (green for carbon atom, blue for nitrogen atom and red for oxygen atom). The distances of hydrogen bonds, salt bridges formed between substrate atoms and atoms from active residues are labeled.

Docking four aliphatic polyamines, hydrazine and histamine (see chemical structures in Fig 2) in the crystal structure active site revealed by the structure demonstrated that spermine is a good ligand showing the highest binding affinity (-5.7 kcal/mol), agmatine, spermidine, histamine, hexamethylenediamine, and putrescine affinity energies of 5.4, 5.0, 4.7, 4.5, 3.8 kcal/mol, respectively, which is basically in consistent with the experimental results. All the test molecules are docked in the active center. The docking solution for all molecules was selected for a further molecular dynamics simulation. It shows that all substrate and enzyme complexes are stable, and spermine and aaNAT5b/SAT complex is most stable (Figs 3, 4 & 5). The interactions of mosquito SAT with its substrates are shown in Fig 3. Spermine interacts with Asp166, Val129, His130, Ser52, Asp48, Tyr28, Glu32 and Thr168 by hydrongen bonding and Tyr29, Phe51, Ile34, Trp101, Leu108, Val129, Ile131, Leu132, Gly165, Phe167, and Ser172 with hydrophobic interactions (Fig 6A). Agmatine, spermidine, histamine, hexamethylenediamine, and putrescine share similar binding residues with spimine, but the enzyme has less interacting residues with them. The detailed binding sites of all tested substrates are shown in Fig 6B, 6C, 6D, 6E, 6F & 6G.

### Gene expression analysis of aaNAT5b

**Fig 7:**
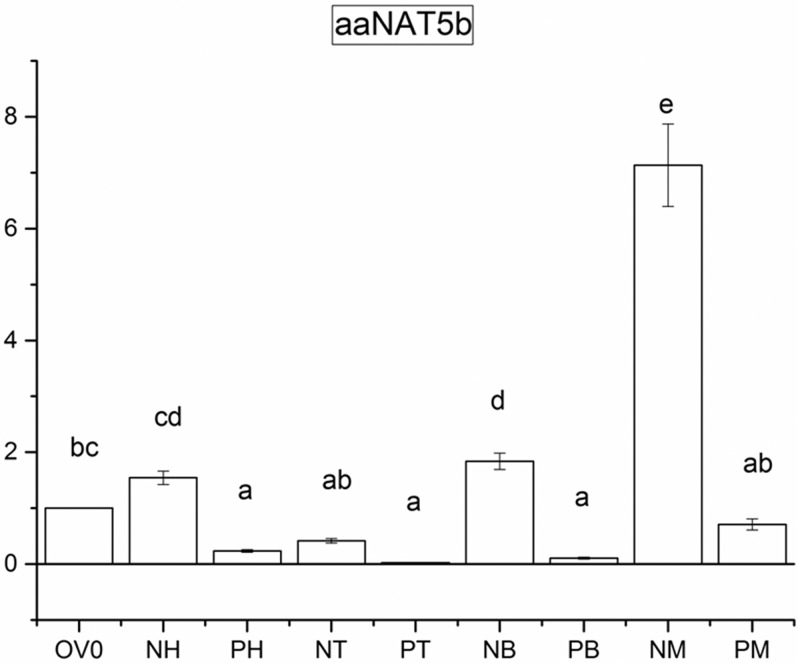
Relative transcriptional levels of aaNAT5b amplified in sugar fed and blood-fed tissues from female adult mosquitoes by qRT-PCR. Each substrate was tested in triplicate. OV0 is ovary sample taken in 48 hour after sugar feeding, and its relative expression quantity was set to 1 unit. X-axis shows adult female blood-fed and non blood-fed tissues, and y-axis shows the relative expression quantity (multiple of OV0) of aaNAT5b. NH, NT, NB, and NM represent head, thorax, abdomen, and midgut of sugar-fed mosquitoes, respectively; PH, PT, PB, and PM represent head, thorax, abdomen, and midgut from blood-fed mosquitoes, respectively. Different letters (a, b, c, d, e and f) indicate statistically significant differences (P< 0.05).

aaNAT5b transcripts were detected in sugar fed and blood fed mosquitoes. aaNAT5b transcripts were found in heads, thoraxes, and abdomens, however they were more abundant in sugar fed mosquitoes than blood fed mosquitoes in each part of the body, particularly in the midgut (Fig 7).

## Discussion

In this report we identified that aaNAT5b is a SAT enzyme in the yellow fever mosquito *Ae*. *aegypti*. Structural analysis of the mosquito SAT revealed the substrate binding sites by experimental and computational approaches. The previous identification of a polyamine N-acetyltransferase-like group of sequences in the available genome sequences for *Ae*. *aegypti* mosquito [8,25] allowed us to select aaNAT5b at first for further biochemical and structural analyses. Although aaNAT5b shares only 8% sequence identity with human SAT, they have similar biochemical function, i.e. both acetylate spermine. Structurally, they both have motifs A and B, which are characteristics of *N*-acyltransferase superfamily proteins [21] and an acetyl-CoA binding sequence folding similar for the cofactor binding (Fig 1B). However, noticeable differences in substrate binding region and biological assembly were seen between two structures. Human SAT forms a dimer and the mosquito SAT is a monomer; mosquito SAT has a disulphide bond, and human SAT does not; and the conformation around the substrate binding sites is different between mosquito (two helixes) and human (one β strand) (Fig 1B), which may explain the substrate specificity of the individual enzymes.

The biological significance of the mosquito SAT is an interesting topic. Our data suggest there are several possible functions of the enzyme in *Ae*. *aegypti*. In terms of their biochemical activity, one possible role for the mosquito SAT may be connected to polyamine metabolism. The yellow fever mosquito *Ae*. *aegypti* is a major vector for dengue virus, CHIKV and ZIKV, it has been reported that CHIKV and ZIKV replications need polyamines [4]. Therefore, mosquito SAT may act as a viral restriction factor of CHIKV and ZIKV by being involved in polyamine acetylation to deplete polyamines. The qRT-PCR results are in consistent with this speculation. RNA level of SAT was lower in bood fed mosquitoes than sugar fed mosquito, particularly in the midgut where viral replication takes place, suggesting polyamines in the mosquitoes are not depleted by SAT after blood feeding, and available for CHIKV and ZIKV replications. Another possible function of the enzyme may be associated with the neurotransmitter, histamine inactivation. This possibility is supported by the observations that it is able to acetylate histamine, which has not been found to be oxidized by any histamine oxidase in insects. An enzyme, AANATL7 from *D*. *melan*ogaster that catalyzes histamine acetylation was also proposed to be involved in histamine inactivation [26].

Although the available genome sequences for a number of insect species make it possible to find a function protein via a bioinformatic approach, it is not the case for identifying mosquito SAT without further biochemical investigation because there was no adequate biochemical and structural data to be used to predict SAT sequence in mosquitoes. Herein, we provided the identification and structural information for an *Ae*. *aegypti* SAT, e.g. substrate binding residues, which could be used for predicting more SAT in mosquitoes and other insects.
